# A Rare Case of Amyloidosis of the Eyelid and Conjunctiva

**DOI:** 10.1155/2016/2915196

**Published:** 2016-09-26

**Authors:** Zaria Ali, Bertie Fernando

**Affiliations:** ^1^Manchester Royal Eye Hospital, Oxford Road, Manchester M13 9WL, UK; ^2^Department of Ophthalmology, East Lancashire Teaching Hospitals, Burnley General Hospital, Casterton Avenue, Burnley BB10 2PQ, UK

## Abstract

Amyloidosis of the eyelid is uncommon and is typically associated with systemic associations. In contrast, amyloidosis of the conjunctiva is often localised with no other associations. We present a rare case of a 92-year-old gentleman with both cutaneous lid lesions and conjunctival amyloid with no systemic involvement. Biopsy demonstrated the hallmarks of amyloid and treatment has remained conservative. He remains at the department to be monitored for secondary glaucoma.

## 1. Introduction

Amyloidosis is a complex disorder involving the deposition of abnormally folded proteins which can affect various areas of the body including the orbit [1–3]. It can present with a diverse array of symptoms depending on which organs are affected [4]. Classification has changed from primary and secondary amyloidosis to light chain amyloidosis and amyloid A protein amyloidosis [1]. It is important clinically to further classify amyloidosis into localised or systemic as systemic amyloidosis can prove to be fatal [4].

Conjunctival and lid amyloidosis is particularly rare [1, 5, 6]. When the lid is affected, it is often found to be associated with systemic conditions [6–8]. In contrast, amyloidosis of the conjunctiva is often localised with no other associations [5, 8]. We present a rare case of a 92- year-old gentleman with both cutaneous lid lesions and conjunctival amyloid with no systemic involvement.

## 2. Case Report

A 92-year-old gentleman presented with an area of inflammation affecting his upper and lower right eyelid. He was initially referred to exclude blepharitis. He had a history of extensive macular degeneration bilaterally which had been treated previously with intravitreal injections, and he is registered as partially sighted. He was otherwise healthy.

**Figure 1:**
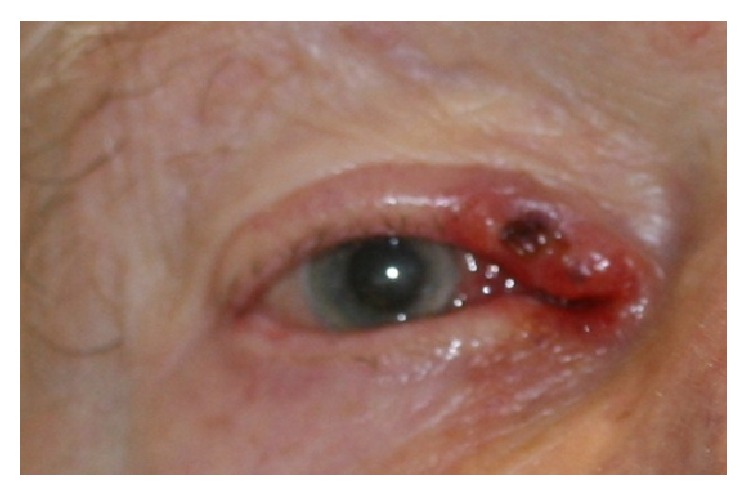
Nodular ulcerative lesion noted to right lid margin.

**Figure 2:**
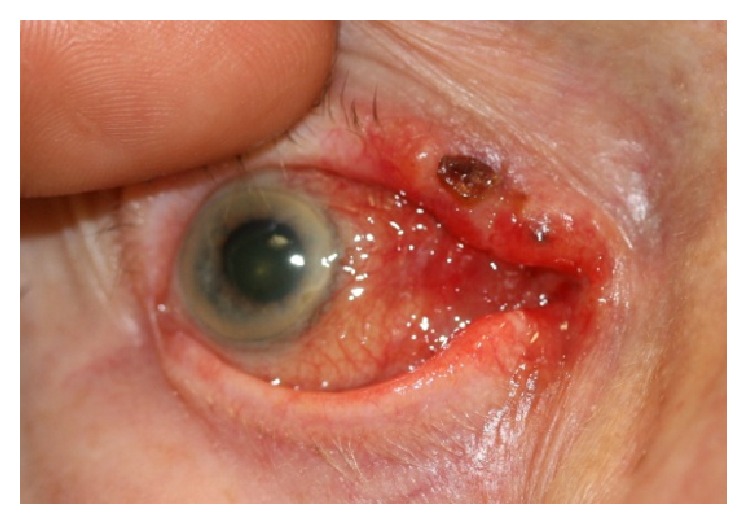
Thickened conjunctiva and caruncle showing red deposits.

On examination, visual acuity was hand movements in the left due to a retinal scar and 6/60 in the right. Early signs of cataract were found in the right eye. He was noted to have a nodular ulcerative lesion affecting his right lid margins along with the medial canthal area (Figure 1). It was noted that the conjunctiva and caruncle were also thickened (Figure 2). The lateral edge was indistinct, so a punch biopsy was arranged to delineate the extent of the lesion. The clinical suspicion was that of an infiltrative basal cell carcinoma.

Punch biopsy of the eyelid lesion along with incisional biopsies of the conjunctival lesions was carried out. All three specimens showed similar features including pieces of tissue covered by atrophic and attenuated epithelium. The underlying stroma showed amorphous eosinophilic fragmented material. Stains were carried out which showed apple-green birefringence on polarised light with Congo red stain. The appearances were those of amyloidosis. There was no evidence of carcinoma.

He was referred to a general physician for investigation of systemic associations. All investigations were unremarkable.

He remains under review in the department for secondary glaucoma.

## 3. Discussion

Amyloidosis is characterised by misfolded proteins which are deposited within extracellular space in various tissues and organs, including the orbit [1–3]. It can be both systemic and localised [1, 7]. Amyloidosis of the conjunctiva and eyelid is a rare entity that is typically benign [5, 6, 9]. Preceding causes for amyloidosis can include trauma, infection, and inflammation [5].

When confined to the conjunctiva the amyloid tends to be localised, whereas cutaneous lesions are characteristically associated with systemic disease [1, 7, 8]. Interestingly, our case showed evidence of both lid and conjunctival amyloid but no systemic associations. This is similar to the rare care reported by Pelton et al. [8].

Patients may present with general eye discomfort, stickiness of the eye, or lid deformity [1, 6, 7, 10]. Rarely, it can present with something as innocuous as subconjunctival haemorrhage, due to friability of amyloid deposits [3, 11, 12].

Identification early on is difficult as the characteristic waxy yellow or red lesions which bleed do not appear till later in its clinical course [1, 10].

Biopsy is indicated to rule out malignancy [1, 7]. After Congo red staining, amyloid typically shows apple-green birefringence when examined with light microscopy [1, 4, 13].

Systemic examination and investigations are required to rule out both systemic amyloid and also neoplastic plasma cell disease [4, 9].

The mainstay of treatment appears to be lubricants and steroids to control symptoms [2, 10]. Surgical intervention has proved controversial due to risk of recurrence and haemorrhage; however, there are reports demonstrating excision including en bloc removal as a treatment [3]. Monitoring for secondary glaucoma is recommended as the amyloid deposits can infiltrate the trabecular meshwork [14]. Secondary glaucoma in these cases responds poorly to medical treatment, and surgery may be tentatively used as in refractive cases [15], but there is not much evidence to support this approach.
